# CD4 response, lipid changes and liver outcome in 506 patients receiving nevirapine-based regimens for a median of 9 years

**DOI:** 10.1186/1758-2652-13-S4-P81

**Published:** 2010-11-08

**Authors:** D Podzamczer, JM Tiraboschi, J Mallolas, MA Cárdenes, E Casas, A Castro, S Echevarria, M Leal, JC Lopez Bernanldo de Quirós, S Moreno, T Puig, E Ribera, C Villalonga, JL Gomez Sirvent, JA Garcia Heranejos, J Lopez-Aldeguer, P Barrufet, L Force, I Santos, J Sanz

**Affiliations:** 1 Hospital Universitari de Bellvitge, HIV Unit, Barcelona, Spain; 2 Hospital Universitari de Bellvitge, Edificio Antigua Escuela de Enfermeria 3° planta, Barcelona, Spain; 3 Hospital Clinic de Barcelona, Barcelona, Spain; 4 Hospital Universitario de Canarias, Tenerife, Spain; 5 Hospital Alcalá de Henares, Madrid, Spain; 6 Hospital Universitario de La Coruña, La Coruña, Spain; 7 Hospital de Valdecilla, Santander, Spain; 8 Hospital Virgen del Rocio, Sevilla, Spain; 9 Hospital Gregorio Marañón, Madrid, Spain; 10 Hospital Ramón y Cajal, Madrid, Spain; 11 Hospital Arnau de Vilanova, Lleida, Spain; 12 Hospital Vall dÂ´Hebron, Barcelona, Spain; 13 Hospital Son Dureta, Palma de Mallorca, Spain; 14 Hospital Dr. Negrin, Las Palmas, Spain; 15 Hospital Santa M° del Rosell, Murcia, Spain; 16 Hospital La Fe, Valencia, Spain; 17 Hospital de Mataró, Mataró, Spain; 18 Hospital de la Princesa, Madrid, Spain

## Purpose of the study

To evaluate long-term outcomes in pts maintaining a NVP-based regimen

## Methods

Retrospective cohort study. Pts received a NVP regimen for at least 5 years and continued up to present. Demographic, clinical, and analytical variables were recorded. A sample size of 506 pts was randomly selected from participating cohorts

## Summary of results

Median follow up 8.9 (5.7-11.3) years (506 pts followed ≥6 years and 270 ≥ 9 years). At baseline: 74% men, 47 years old, 36% drug users, 40% AIDS, 40% HCV+, 14% alcohol. 45% detectable VL, CD4 395/uL, 19% CD4 < 200/uL, 27% ALT Grade 1-2, 36% AST grade 1-2. 30% ART naïve. 84% received NVP+2 nucleosides (NRTI) during the study period, 17% PI.

Most frequent current combinations were NVP+TDF/FTC in 31%, +ABC/3TC in 24% and +ZDV/3TC in 22%. 97% reached undetectable VL. In pts receiving 2 NRTI+NVP (n=423), regardless of being HCV+ or -, a significant increase was observed in general health status markers: hemoglobin, platelets and albumin (all p<0.001), and +218 and +322 CD4 cells increase after 6 and 9 years (p<0.001). Triglyceride levels decreased 19% and total cholesterol 4% in pretreated pts vs 9% and 12% increase in naive pts. After 6 years, the proportion of pts with lipid levels above (below in HDL) the NCEP thresholds for recommending lipid lowering therapy was 50% for TC, 43% TG, 34% LDL and 14% HDL.

Regarding liver outcomes in the 506 pts, a significant decrease in ALT and AST levels were found in naive (p=0.02, p<0.001) and HCV+ pts (p=0.065, p<0.001), while a strong decrease in alkaline phosphatase (AP) levels (up to -44%) was observed in naive and pretreated pts as well as in HCV+ or — (all p<0.001), regardless of TDF use. GGT levels increased by 78% regardless of the patient status (p<0.001).

This favourable changes in liver function tests occurred despite 53% of 89 pts in whom biopsy or fibroscan was performed after a median of 7.1 years of NVP therapy, fibrosis (≥ F2 and/or 7≥kPa) was detected. In addition, as a consequence of transaminase and platelet changes, Fib-4 index significantly decreased in ARV naive HIV/HCV pts at 9 years (p=0.01)

## Conclusions

Patients receiving a long-term NVP-including regimen, show a progressive improvement in general health status and CD4 response, an acceptable lipid profile and favourable changes in liver function tests, even in those with HCV+. The marked decrease in AP levels shown in this large cohort of NVP-treated pts merits further study.

